# Sequential trafficking of Env and Gag to HIV-1 T cell virological synapses revealed by live imaging

**DOI:** 10.1186/s12977-019-0464-3

**Published:** 2019-01-15

**Authors:** Lili Wang, Sudeh Izadmehr, Edwin Kamau, Xiang-Peng Kong, Benjamin K. Chen

**Affiliations:** 1 Department of Medicine, Division of Infectious Diseases, Immunology Institute, Mount Sinai School of Medicine, New York, NY 10029 USA; 2 Department of Biochemistry and Molecular Pharmacology, New York University School of Medicine, 550 First Avenue, New York, NY 10016 USA

**Keywords:** HIV-1, Virological synapse, Envelope protein, Gag, GFP

## Abstract

**Background:**

HIV infection is enhanced by cell adhesions that form between infected and uninfected T cells called virological synapses (VS). VS are initiated by an interaction between Env and CD4 on cell surfaces and result in the recruitment of virus assembly to the site of cell–cell contact. However, the recruitment of Env to the VS and its relationship to Gag recruitment is not well defined.

**Results:**

To study the trafficking of HIV-1 Env through the VS, we constructed a molecular clone of HIV carrying a green fluorescent protein-Env fusion protein called, HIV Env-isfGFP-∆V1V2. The Env-isfGFP-∆V1V2 fusion protein does not produce virus particles on its own, but can be rescued by cotransfection with full-length HIV constructs and produce virus particles that package the fluorescent Env. These rescued fluorescent Env can participate in VS formation and can be used to directly image CD4-dependent Env transfer across VS from donor to target cells. The movements of fluorescently tagged Gag and Env to the VS and transfer into target cells can be also tracked through live imaging. Time lapse live imaging reveals evidence of limited Env accumulation at the site of cell–cell contact shortly after cell adhesion, followed by Gag re-distribution to contact area. Both Gag and Env can be recruited to form button-like spots characteristic of VS.

**Conclusions:**

Env and Gag are recruited to the VS in a coordinated temporal sequence and subsequently transfer together across the synapse into the target cell. Env accumulations, when observed, are earlier than Gag re-distribution to the contact area during formation of VS.

**Electronic supplementary material:**

The online version of this article (10.1186/s12977-019-0464-3) contains supplementary material, which is available to authorized users.

## Background

HIV-1 dissemination through cell–cell contacts called virological synapses (VS) is an efficient means of viral propagation that is relevant to viral spread in vitro and in vivo [1–3]. The formation of VS is triggered by the interaction of envelope protein on infected cells and CD4 on target cells [4]. During HIV transmission across VS, HIV-1 Gag, Env and CD4 were found to localize at the site of cell–cell contact in an actin-dependent manner [1]. Antibodies against Env or CD4 block the adhesion of infected and uninfected cells [5]. Recruitment of Gag to the VS and transfer through the VS has been observed to occur following cell-adhesion capable of forming large platforms of viral assembly referred to as synaptic buttons [6]. However, the pattern of recruitment of Env to VS, and its relationship to Gag recruitment is largely uncharacterized due to the challenges of identifying non-disruptive methods to fluorescently label Env.

Fluorescent labeling of Env using enzymatically-coupled fluorophores has been employed to measure the conformational state of Env with single molecule FRET on virus particles [7]. However, these methods are well suited to track Env trafficking inside living cells. We undertook the challenges of generating a functional fluorescent fusion protein with HIV Env. Env is a trimer of heterodimers and each protomer forms multiple internal disulfide bonds that greatly limit the locations that can tolerate the insertion of a fluorescent protein. In addition, the Env trimer is metastable and fluctuates between different conformational states when it engages the CD4 receptor or CD4-bound conformation inducing antibodies [8–10]. The insertion of large fluorescent protein into Env can enable fluorescent imaging studies, yet has the potential to impact important functional states of Env. Here our goal was to design a fusion protein to allow tracking of Env recruitment to the VS and to observe its relationship to virus particle biogenesis with minimal disruption of its functional states. Insertion of GFP at the N-or C-terminal may adversely affect synthesis of Env in the endoplasmic reticulum or packaging onto virus particles, respectively. In an effort to preserve Env structure and function, we adopted the strategy of substituting the V1V2 domains with GFP. V1V2 domains can be dispensable for infection [11, 12] and insertion of short peptide tags are tolerated at this site [13]. To minimize misfolding of the fluorescent protein we employed a highly-stable allele of GFP called superfolder GFP (sfGFP) [14]. We removed the entire V1V2 domain and connected the N and C termini of superfolder GFP to the common V1V2 loop base. We studied the utility of this GFP-tagged virus to follow Env trafficking. Here we report the development of an HIV-1 clone, HIV Env-isfGFP-∆V1V2, which enables live monitoring of the transfer of virus between cells and distribution of Env before, during and after VS formation. Simultaneous imaging of fluorescent Gag and Env fusion proteins during VS formation revealed independent movements of the proteins prior to synapse formation, and support a model for sequential ordered accumulation following cell–cell adhesion where the presence of Env was detected prior to Gag accumulation.

## Results

### Construction of an HIV clone with GFP-tagged Env

**Fig. 1:**
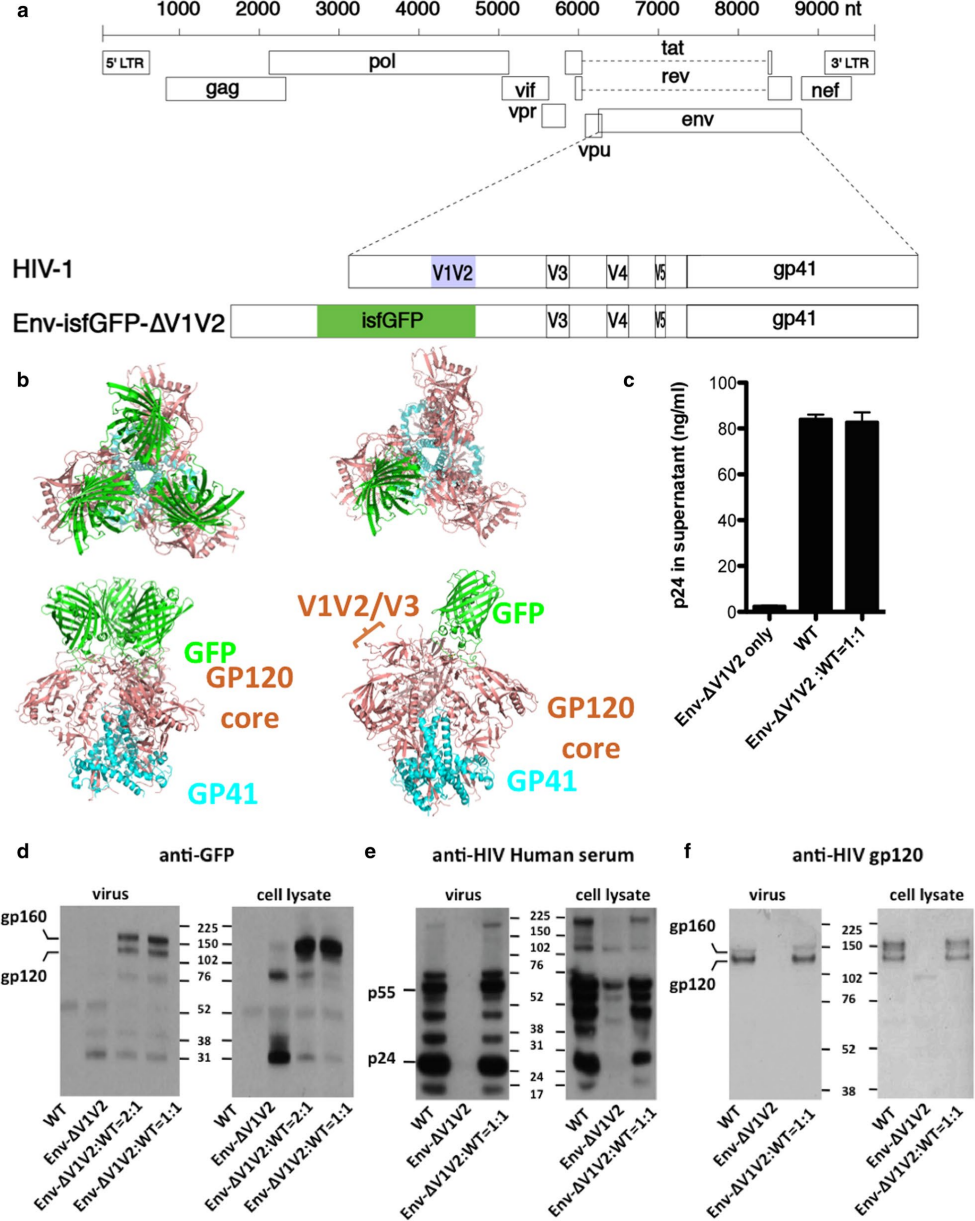
Construction and expression of proviral clone HIV Env-isfGFP-∆V1V2. **a** Map of superfolder GFP insertion into HIV-1 (NL4-3) replacing the V1V2 domain of Env. **b** Modeling of Env trimers. Upper left: top view of Env-isfGFP-∆V1V2 homotrimer. Lower left: side view of Env-isfGFP-∆V1V2 homotrimer. Upper right: top view of a 2:1 Env: Env-isfGFP-∆V1V2 heterotrimer with one Env-isfGFP protomer. Lower right: side view of the Env-isfGFP-∆V1V2 heterotrimer. **c** Viral production was determined by p24 ELISA of supernatants from provirus transfected 293T cells. Deficiency of Env-isfGFP-∆V1V2 particle production was complemented by co-transfection of wild-type HIV NL4-3. Cell lysates or purified supernatant virions from provirus transfected 293T cells were examined by Western Blot using an anti-GFP antibody (**d**), anti-HIV human serum (**e**) or anti-gp120 antibody (**f**). Supernatant virions were purified through a 20% sucrose cushion from 293T-transfection supernatants. Both cell lysates and viral supernatants were collected at 48 h post transfection

To enable live imaging of the HIV Env glycoprotein during HIV infection and cell-to-cell HIV spread, we inserted the GFP into *env* within a full-length molecular clone of HIV, NL4-3. As a strategy to minimize the disruption of Env trafficking signals, a superfolder allele of GFP [14], sfGFP, was inserted directly into HIV-1 envelope coding sequences to replace the V1V2 domain of Env (Fig. 1a). The N and C termini of the GFP beta barrel were connected to the base of the V1V2 loops. A 3D-model was generated for both the homotrimer and heterotrimer configurations of Env with sfGFP inserted. A disulfide bond was modeled into sfGFP to place it more accurately into the trimer. Modeling an Env trimer with three sfGFPs (Fig. 1b) indicated the possibility of steric clashes between the GFP monomers, however these steric clashes were not apparent when one sfGFP is placed in the heterotrimer with two wild-type Env. Given the potential for steric clashes between homotrimers Env might interfere with its expression or function, we anticipated that we might need to complement Env-sfGFP-∆V1V2 with wild type Env to generate functional Env heterotrimers. Since the V1V2 domain is not required to generate infectious virus [11, 12], it is also plausible that insertion of GFP into the base of this peptide loop could be compatible with infectivity.

### Env-isfGFP-∆V1V2 protein is produced when complemented by wild-type HIV-1

Transfection of the HIV Env-isfGFP-∆V1V2 into 293T cells or Jurkat cells revealed a green fluorescent signal (Additional file 1: Fig. S1), but this mutation in Env exhibited poor overall viral gene expression, including low levels of Gag expression and virus production (Fig. 1c). Co-expression of wild-type NL4-3 rescued the production of viral particles (Fig. 1c) and was compatible with Env-isfGFP-∆V1V2 fluorescence (Additional file 1: Fig. S1). A Western blot to detect the GFP-Env fusion protein in cells transfected with HIV Env-isfGFP-∆V1V2 indicated that single transfection of HIV Env-isfGFP-∆V1V2 fails to produce full length Env. Only small fragments of GFP were observed in the cell lysates (Fig. 1d). However, when co-transfected with wild-type HIV, the expression of the full-length Env-isfGFP-∆V1V2 protein was rescued. A Western blot to detect HIV-1 proteins with a polyclonal pooled anti-HIV-1 IgG revealed very weak expression of Gag in cell lysates and viral supernatant of HIV Env-isfGFP-∆V1V2 single transfectants. The co-expression of wild-type HIV rescued HIV Env-isfGFP-∆V1V2 expression and resulted in Gag expression and viral production to wild-type levels (Fig. 1e). Western blot with anti-gp120 revealed that the size of Env with sfGFP substitution was similar to that of wild-type Env (Fig. 1f). Although the V1V2 loop is much smaller than GFP, the similar mass of the WT and fusion protein may be attributed to replacement of the richly glycosylated V1V2 loop with an unglycosylated GFP.

### Superfolder GFP envelope is incorporated into viral particles after complementation

**Fig. 2:**
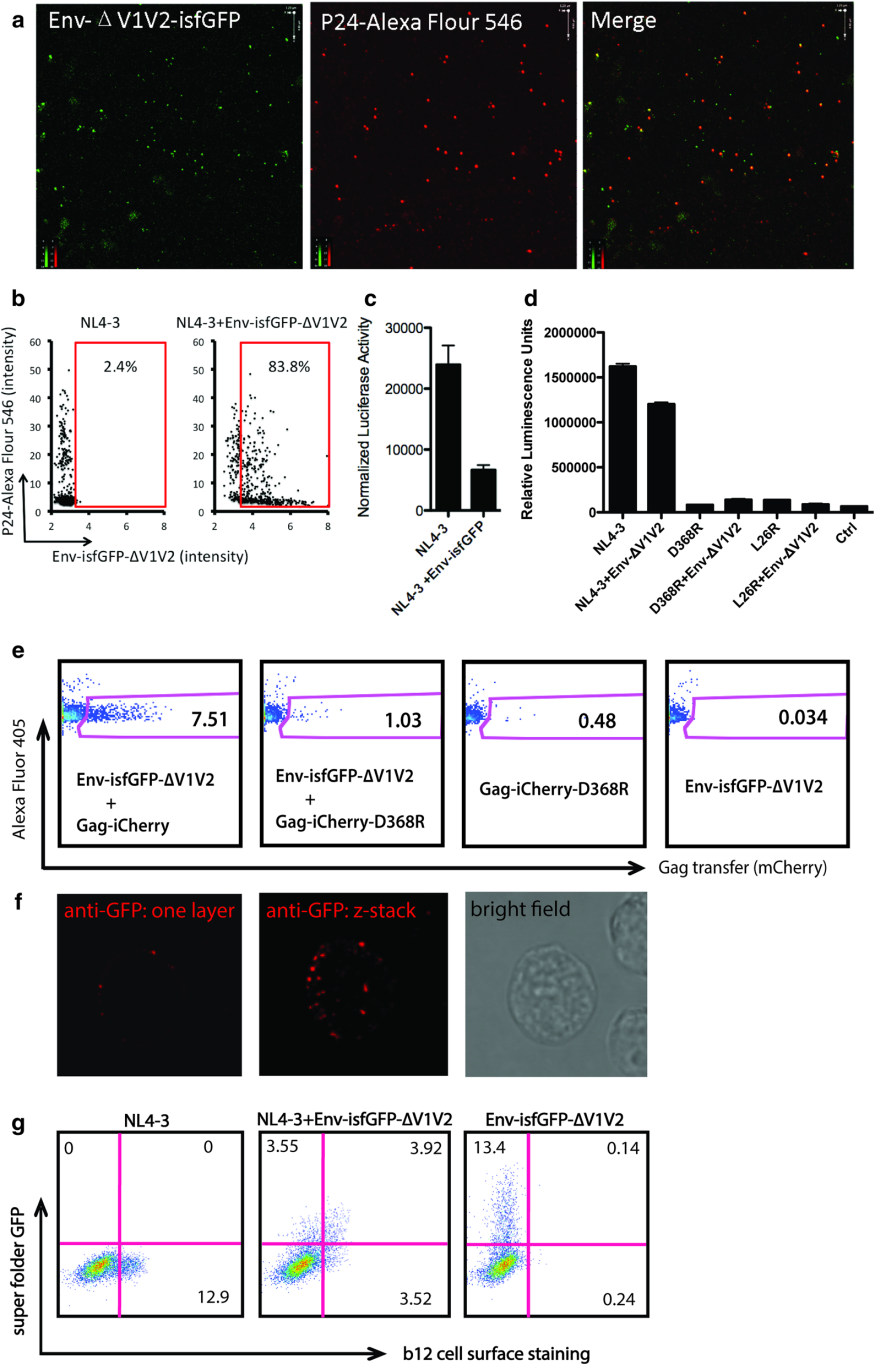
Env-isfGFP-∆V1V2 can be packaged onto virus particles when co-expressed with wild-type Env but the particles have decreased infectivity. **a** Fluorescence imaging of viral particles purified from supernatants of NL4-3 and Env-isfGFP-∆V1V2 co-transfected 293T cells. Purified viruses were attached to poly-lysine treated coverslip, fixed, permeabilized and then stained by anti-p24 primary followed by Alexa Fluor 546 conjugated secondary antibody. **b** Two-color fluorescence plot of fluorescent HIV particles from NL4-3 or NL4-3 Env-isfGFP-∆V1V2 co-transfectanted with NL4-3. The images of purified virus particles acquired as in (**a**) were segmented using Imaris software and fluorescence intensity associated with Gag and Env is plotted. The gates show proportion of particles with relative sfGFP intensity > 3.2 fluorescence units. **c** Infectivity of supernatant viruses by Tzm-bl assay normalized by p24 ELISA. **d** Infectivity of Env-isfGFP-∆V1V2 when complemented with mutant HIV Env constructs. Infectivity of the same volume supernatants from indicated co-transfected cells was determined by Tzm-bl assay. **e** Cell-to-cell transfer of HIV Env-isfGFP-∆V1V2 requires a CD4 binding competent Env. Jurkat cells were nucleofected with indicated constructs or combination of constructs, 24 h after nucleofection, they were co-cultured with primary CD4^+^ cells, the gates show transfer of Gag to primary CD4^+^ cells. **f** Cell surface GFP staining of cells coexpressing Env-isfGFP-∆V1V2 and WT NL4-3. Cells were stained at 4 °C with anti-GFP antibody and AF564 secondary antibody before fixation. The red dots show sfGFP Env on cell surface. **g** Cell surface Env can be stained by b12, which recognizes the CD4 binding site on Env

To examine further whether envelope protein carrying an sfGFP fusion protein is incorporated into viral particles, we performed immunostaining on purified viral particles. Detection of the viral capsid protein p24 with Alexa Fluor 546 was visualized with the green fluorescence of Env-isfGFP-∆V1V2 to assess the efficiency of packaging of the chimeric Env onto virus particles. The chimeric virus was observed in the cell culture supernatant of cells co-transfected with HIV Env-isfGFP-∆V1V2 and an HIV-1 construct with wild-type envelope (Fig. 2a). Puncta that were red and green represent viral particles that incorporated Env-isfGFP-∆V1V2 onto virus particles stained with the AlexaFluor 546. Single red and single green puncta were also visualized, which represent independently released Gag or Env antigens in the preparation. The proportion of viruses incorporating Env-isfGFP-∆V1V2 as a fraction of all the viruses produced by co-transfection was measured using software based image segmentation (Imaris). Compared to wild-type Env expressing viral particles, co-transfected cells produce viral particles that displayed an obvious shift in GFP fluorescence indicative of associated Env-isfGFP-∆V1V2 (Fig. 2b). The GFP signal on these particles is relatively low compared to Gag-iCherry-associated Cherry fluorescence, consistent with the lower number of Env molecules on viral particles [15, 16]. Compared to non-GFP tagged wild type particles, viral particles with sfGFP had a relative fluorescence intensity larger than 3.2 relative fluorescence units, so we “gated” particles at this intensity calculate the fraction of particles that packaged the fluorescently tagged Env. Based on this calculation, 83.8% of viral particles produced by co-transfection had incorporated detectable Env-isfGFP-∆V1V2 (Fig. 2b).

### Env-isfGFP-∆V1V2 is compatible with infectivity and cell-to-cell viral transfer

We next assessed whether these chimeric viruses were infectious on the HIV indicator cell line, TZM-bl. The TZM-bl assay indicated that the co-transfected wild-type HIV and HIV Env-isfGFP-∆V1V2 virus particles display 32.4% ± 5 infectivity compared to wild-type HIV virus (Fig. 2c). The results suggest that chimeric viruses produced by NL4-3 and Env-isfGFP-∆V1V2 co-transfection are compatible with infectivity of the virus but with decreased infectivity for cell-free infection.

Prior studies have indicated that cooperative subunit interactions are a general feature of the Env trimer: one defective protomer can cooperatively interact to trigger the functional determinants on adjacent protomer(s) [17, 18]. The D368R point mutation disrupts the interaction between CD4 and envelope protein. The D368R NL4-3 viruses and chimeric viruses from D368R NL4-3 plus Env-isfGFP-∆V1V2 co-transfectant were not infectious in the form of cell-free virus particles (Fig. 2d). However, when performing cell-to-cell transfer, Env-isfGFP-∆V1V2 was partially complemented by coexpression with the D368R mutant and showed a low level of viral antigen transfer to target cells (Fig. 2e). Considering D368R is deficient in CD4 binding, we conclude that the Env-isfGFP-∆V1V2 is partly capable of interacting with CD4 and responsible for the viral antigen transfer. The viruses carrying Env-isfGFP-∆V1V2 complemented with D368R Env were not infectious, suggesting that the GFP insertion into Env is not compatible with fusion in a mixed heterotrimer. As a result, we infer that Env-isfGFP-∆V1V2 is able to form a VS through its own CD4 binding function but not able to induce virus particle fusion. In wild type-HIV-complemented viral particles, the rescue of infectivity may be due to the WT Env of a mixed heterotrimer, or in WT homotrimers.

In cells expressing Env-isfGFP-∆V1V2 complemented with a WT Env, we detected the GFP inserted into Env at the cells surface using an anti-GFP antibody followed by fluorescent secondary antibodies. This indicates that the Env-sfGFP fusion protein is expressed on the surface of the cells (Fig. 2f and Additional file 1: Figure S2). We further tested surface expression with the anti-Env monoclonal antibody b12 (CD4 binding site broadly-neutralizing antibody), and observed that the Env CD4bs epitope is expressed on the surface when EnvisfGFP-∆V1V2 Env is complemented with WT Env (Fig. 2g). Based on above results, we proceeded to study cells cotransfected with Env-isfGFP-∆V1V2 and WT Env HIV to track Env during the formation of VS and viral antigen transfer.

### Expression pattern and kinetics of Env-isfGFP-∆V1V2

**Fig. 3:**
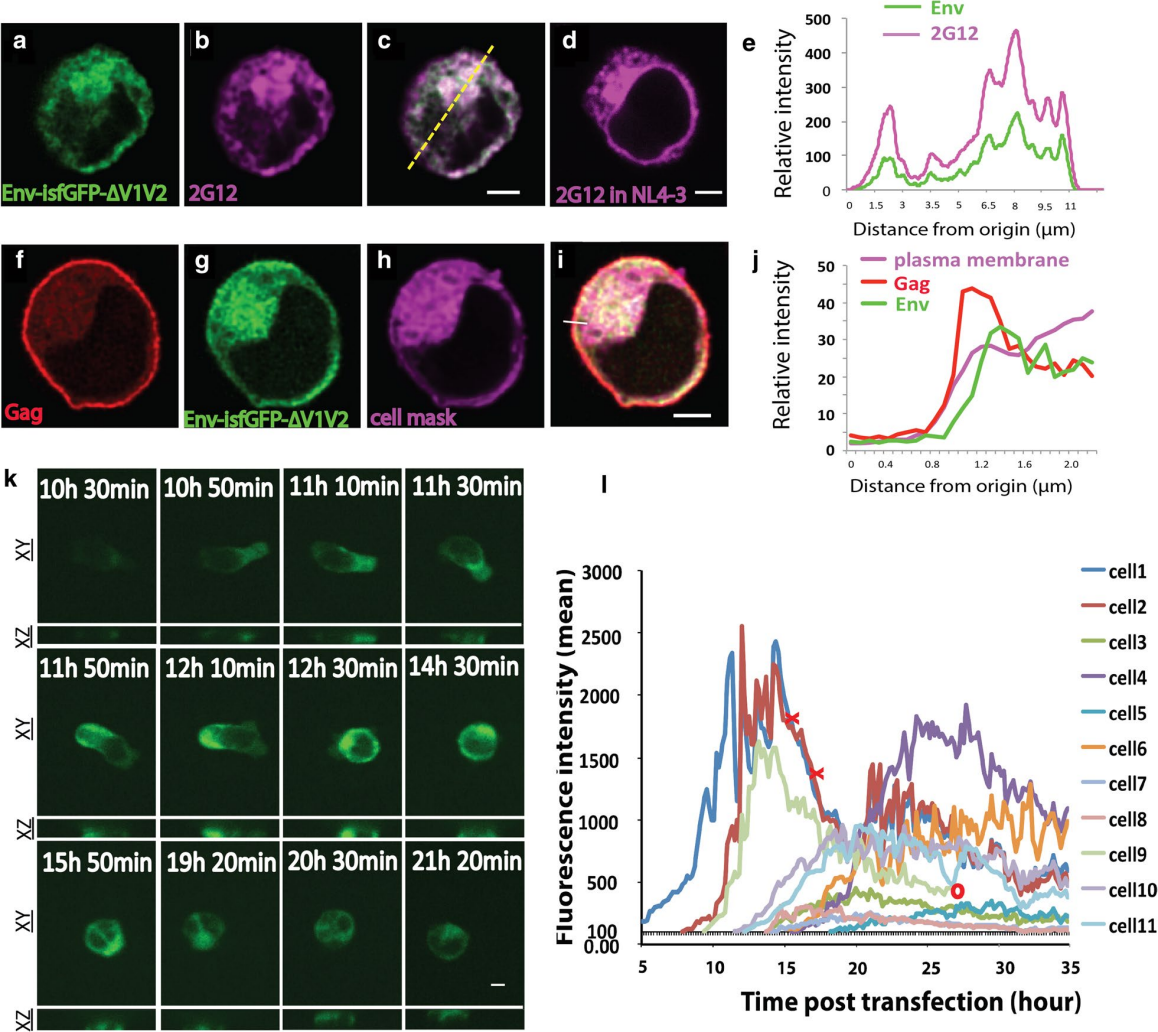
Fluorescent images of Env-isfGFP-∆V1V2 in lymphoblastoid cell line, Jurkat E6. Jurkat cell transfected Env-isfGFP-∆V1V2 plus NL4-3 (**a**–**c**), or with NL4-3 only (**d**), or with Env-isfGFP-∆V1V2 plus Gag-iCherry (**f**–**i**) were fixed, permeabilized and stained with anti-Env mAb 2G12 (**a**–**d**) or Cell Mask Deep Red (**f**–**i**). Fluorescence intensity across co-transfected Jurkat cell shown in merged panel of (**c**) and (**i**) were displayed in (**e**) and (**j**). **k** and **l** Live time-lapse confocal fluorescence imaging of an Env-isfGFP-∆V1V2-expressing Jurkat lymphoblastoid T cell. Confocal z stacks were acquired at 10-min intervals from 5 h post transfection for 30 h. **k** Montage of fluorescence images of one cell (cell 9) illustrating changes in the fluorescence pattern of Env-isfGFP-∆V1V2 at indicated time points marked in (**l**). **l** Graph of fluorescence intensity of Env-isfGFP-∆V1V2-expressing cells in one field over time. Mean intensities above background was plotted. The expression of most cells peaked between 20-25 h post transfection. Red X indicates cell death of cell 1 and 2 and O indicates cell 9 moves out of field. Bar = 3 μm

To test the fidelity of Env-isfGFP-∆V1V2 as an indicator for Env localization, we performed immunofluorescence staining of Env-isfGFP-∆V1V2 and compared the patterns to native Env. Using the glycan binding monoclonal antibody 2G12 to detect Env in the cotransfected cells, we observed that the Env-isfGFP-∆V1V2 fluorescence co-localized well with immunostained Env (Fig. 3a–e). The distribution of Env stained with 2G12 in cotransfected cells exhibited a similar distribution to WT Env expressed in WT HIV NL4-3 transfected cells (Fig. 3d). As expected, the predominant signal for Env was found in the intracellular compartments with minimal expression at the cell surface. To assess the relative intensity of Gag and Env across the cell, we measured the intensity of Env expression across a cell expressing Env-isfGFP-∆V1V2 and WT Env and found that the fluorescent Env signal at the cell surface was localized to a region just internal to the Gag layer on the cytoplasmic side (Fig. 3f–j). We next examined the kinetics of de novo expression of Env-isfGFP-∆V1V2: wild-type Env HIV-1 heterotrimers. A time-lapse 3-dimensional confocal fluorescence imaging system was used to acquire images every 10 min from 5 h post transfection for 30 h. Env expression levels in the cell peaked at 20–25 h post transfection, and declined thereafter (Fig. 3l). At an early stage of Env expression, the cell actively moved and changed its shape (Fig. 3k). However, following peak Env expression, the cell rounded and slowed in its movement, perhaps indicative of cell toxicity at later stages of infection. A movie of the *de*-*novo* expression of Env is displayed in Additional file 2: Movie S1.

### Complemented Env-isfGFP-∆V1V2 participates in cell-to-cell transfer

**Fig. 4:**
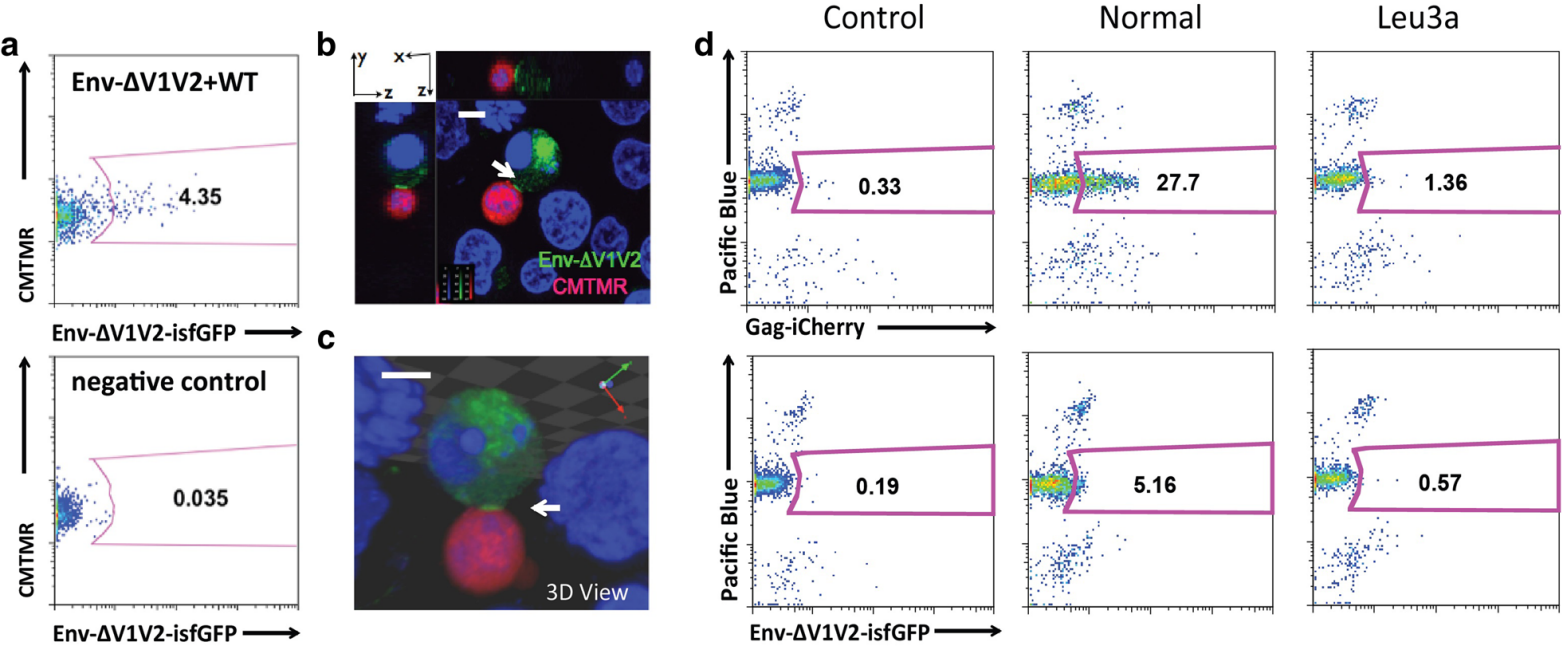
Transfer of fluorescent Envelope from donor to target cells through cell-to-cell spread. **a** Jurkat cells co-transfected with Env-isfGFP-∆V1V2 and NL4-3 were used as donor cells and primary CD4 T cells were used as target cells. Gates indicate the portion of target cell transferred with Env after co-culture. **b** and **c** Co-cultured cells were attached onto poly-lysine treated cover glass, fixed and observed under fluorescent confocal microscope. Bar = 5 μm. **d** Jurkat cell co-transfected with Env-isfGFP-∆V1V2 and Gag-iCherry were used as donor cells. Gates indicate the percentage of target cells carrying transferred Gag or Env after co-culture. Control condition examines donor and target cells maintained in separate wells and fixed before flow cytometry, Leu3a condition tests the inhibitory potential of Env-blocking anti-CD4 antibody Leu3a

When cotransfected with a wild-type virus, HIV Env-isfGFP-∆V1V2 produces fluorescent Env from co-expressing cells. To further test whether the fluorescent Env on these viruses can transfer across VS, we performed a cell-to-cell viral transfer assay. Jurkat cells co-transfected with Env-isfGFP-∆V1V2 and NL4-3 were used as donor cells, and primary CD4 T cells were used as target cells. Co-transfectants retained the ability to engage in cell-to-cell transfer of HIV-1 (Fig. 4a), and the fluorescent envelope protein could be found to accumulate at cell–cell contact area to form a VS button (Fig. 4b, c). These results indicate Env-isfGFP-∆V1V2 may be used to study the distribution of Env during HIV-1 synapse formation and cell-to-cell transfer. To simultaneously follow Gag and Env transfer from donor to target cells, we co-transfected donor Jurkat cells with HIV Gag-iCherry and HIV Env-isfGFP-∆V1V2 and performed a cell-to-cell transfer assay. The Gag-iCherry transferred to 28% of the target cells, and 5% of these target cells also internalized Env-isfGFP-∆V1V2. Given that Env has been estimated to be present in virus particles at 50–100 fold lower levels than Gag [19, 20] the lower frequency of Env signal in target cells is expected. Importantly, we observed that the transfer of fluorescent Gag and Env was blocked by the anti-CD4 antibody that targets the gp120 binding site, Leu3a, indicating that the transfer process is CD4 dependent (Fig. 4d).

### Time lapse observations of Env at the sites of infected cell contact with uninfected cells

**Fig. 5:**
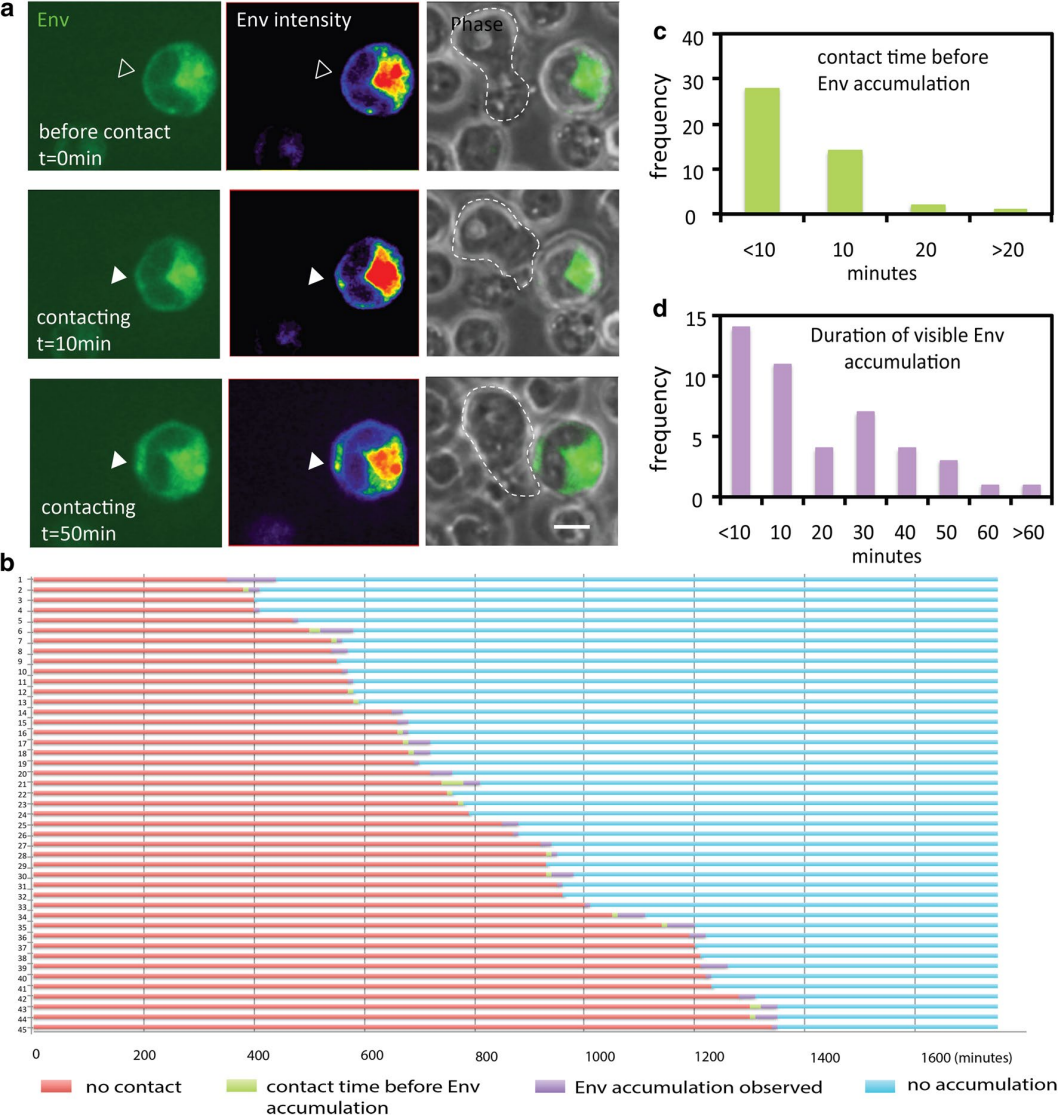
Transient Env accumulation at sites of cell–cell contact. Time-lapse live confocal fluorescence images of Env-isfGFP-∆V1V2 expressing cells were acquired at 10-min intervals from 5 h post transfection for 30 h. **a** A target cell contacting an Env-expressing donor cell at t = 0 min. At 0 min, no Env accumulation was observed at contact area (empty arrow). At t = 10 min, there was some accumulation of Env and at t = 50 min, a visible accumulation of Env was noted. An arrow in each panel highlights the same contact site. Bar = 5 μm. **b** Transient Env accumulation observed in some of the 45 prolonged contact events between infected and uninfected cells. Different colors show different localization of Env during cell contacts. The interval between the initiation of cell and Env accumulation occur is shown as a green line. **c** When Env accumulation was observed (green line in **b**), the cells were counted according to the duration of the contact time before env accumulation. **d** When Env accumulation was observed, cells were counted according to the duration of visible Env accumulation

To examine the organization of synapse initiation, we designed live imaging experiments at different frame rates and durations to follow the spatial and temporal distribution of Gag and Env. Using a frame rate of 1 per 10 min interval for 30 h allowed us to follow changes in Gag and Env localization during synapse initiation (Fig. 5). Transient contacts between infected donor cells and potential target cells were frequent, but mostly occurred without leading to stable adhesion, similar to our previous observations [5]. Donor cells can make multiple transient contacts with other target cells before the formation of a stable contact. In some cell–cell conjugates, accumulation of Env was observed at the contact area, shortly after the physical contact (Fig. 5a and Additional file 3: Movie S2). 33 cells with evidence of contact-induced accumulations were observed among 5 different fields from the same time-lapse experiment (Additional file 1: Table S1). A donor cell with multiple contacts with other target cells displayed multiple Env accumulations at each contact area. Among the 33 cells we observed 45 events of contact-induced Env accumulation. For each event, the cell contact time, time to Env accumulation and Env accumulation duration were recorded (Fig. 5b). Quantification of these contact-induced accumulations revealed that in 42 out of 45 events, some Env recruitment to the contact site was observed within 10 min from the moment of initial contact (defined as t = 0) (Fig. 5c). Analysis of the duration of Env accumulation indicated there are two types of accumulation: one class of Env accumulation lasted no more than 10 min, while another class had an average duration of 30 min. Persistent Env accumulations longer than 60 min at cell–cell contact sites were only rarely observed (Fig. 5d). Since the Env CD4 interaction is required for the initiation of cell–cell adhesions [5], we conclude that the levels of Env recruitment required for synapse formation represent a small fraction of the total Env in the cell.

### Gag and Env traffic sequentially to the VS

Gag can bud from the plasma membrane to form virus-like particles in the absence of other viral proteins or virus RNA [21]. In transfected Jurkat cells, Gag localizes to the plasma membrane before synapse formation [2]. Previous high-speed confocal imaging revealed that much of the movement of Gag to the VS was driven by the redistribution of plasma membrane-associated Gag to the synapse area rather than de novo accumulation [2]. In this study, using spinning disk confocal imaging we observed examples of independent movement of Gag and Env in an HIV transfected cell. In a crawling cell, Gag could be found at the leading edge, where very little Env was found (Additional file 4: Movie S3). In this example Env was predominantly localized in a peri-nuclear pattern. Another acquisition taken at a 10-s frame rate illustrates the recruitment of Gag to a site of cell–cell contact where small puncta of higher concentrations of Env are already present evident when viewed with false-color lookup table. At the beginning of the image series, Gag is distributed evenly on cell membrane while Env is at higher intensity at cell contact site; later Gag is recruited to the contact site and forms a button where Env had previously accumulated (Additional file 5: Movie S4).

**Fig. 6:**
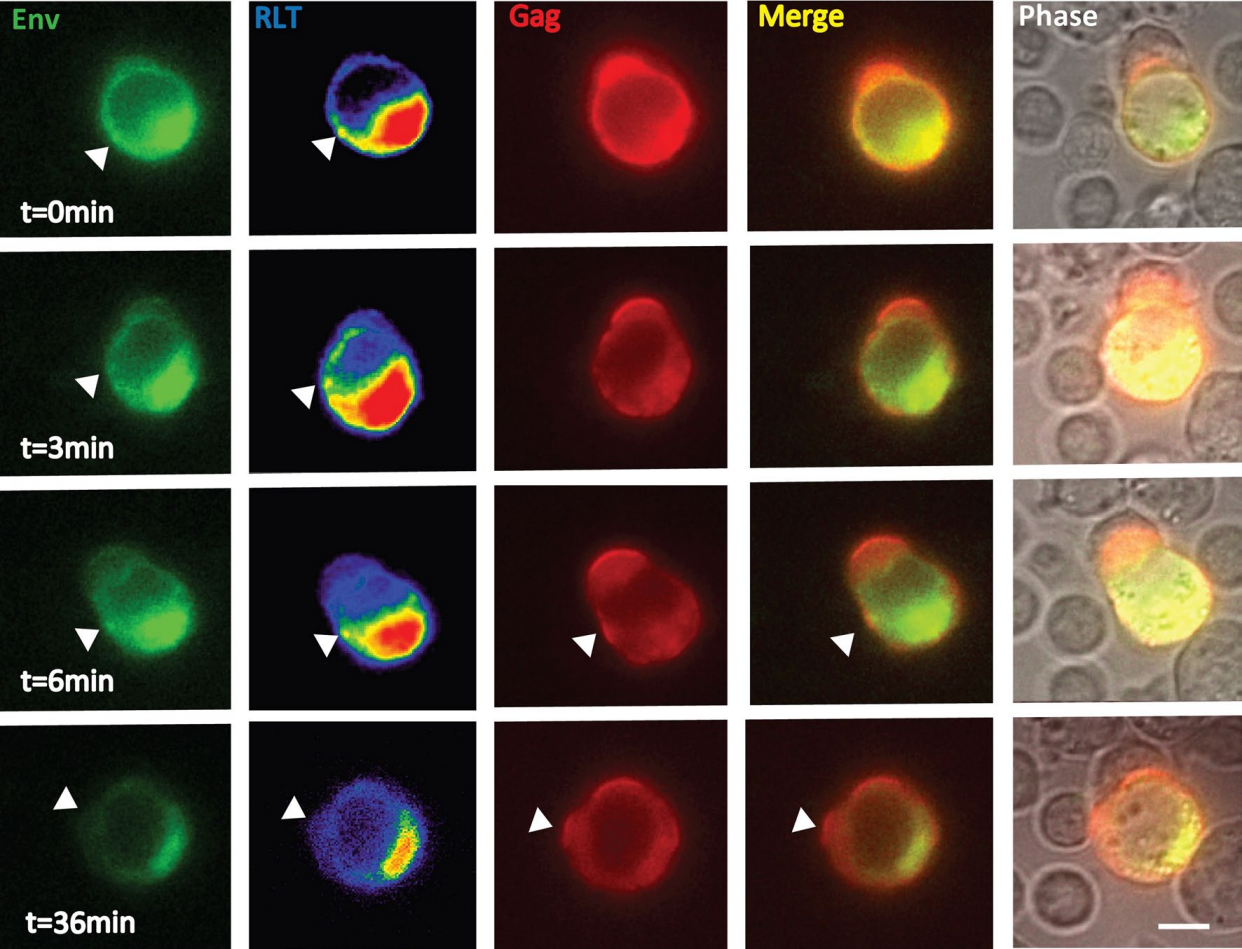
Redistribution of Gag to the virological synapse can be observed after Env accumulation. Time-lapse confocal fluorescence images show the process of VS formation. The donor cell expresses both Gag-iCherry and Env-isfGFP-∆V1V2. Confocal z-stacks were acquired at 3-min intervals from 0.5 h after donor cell and target cell mixing. From 0 to 6 min, donor-target cell adhesion is visualized and Env accumulation was observed (arrow) at the site of cell–cell contact. At t = 0 min, Gag was evenly distributed across the plasma membrane. At t = 3 min, Gag begin to re-distribution and at t = 6 min, increased Gag localization at contact site is evident. At t = 36 min, prominent Gag accumulation at the VS was observed but Env was no longer observed. Independent events observed N = 5. RLT: reference lookup table; Bar = 5 μm

To capture the entire process of VS formation, we acquired videos with a longer frame rate (1 frame every 3 min) for 3 h on a wide-field microscope. Of the 45 HIV-expressing cells in four fields, 16 of them showed co-localization of Gag and Env at the site of cell–cell contact (Additional file 1: Table S2), and five of them putatively captured the whole process of VS formation. During VS formation, we observed examples where Env and Gag were localized sequentially to the cell contact area (Fig. 6, Additional file 1: Figure S3 and Additional file 6: Movie S5). Time-lapse images (Additional file 6: Movie S5) were acquired at a 3-min frame rate and revealed a synapse with sequential recruitment of Env followed by Gag. At 0 min, some Env accumulation was observed at the contact area, while Gag was evenly distributed at the plasma membrane. At 3 min, a small Env accumulation at the contact area was observed with better contrast, (focus on the RLT rendered Env image). At 6 min, Env accumulation at contact area was further enhanced while Gag redistributed to the same area as Env. An organized VS formed at 6 min, when both Env and Gag co-localized at the synapse area. In this example, the relatively high level of Env at the VS was short lived, which may be the result of transfer across the synapse or endocytosis from the cell-surface of the donor cell. At a later time point (36 min), only Gag was observed at the VS, while Env was no longer visible. An additional four sequential accumulations of Env and Gag to the VS were observed on different cell–cell contacts in this study (Additional file 1: Fig. S3). Additional file 7: movie S6 shows one donor cell with five Gag buttons engaged with several target cells simultaneously. In these synapses, Env accumulations are minimally detected above background. We interpret this pattern as potentially being indicative of a later stage of VS formation.

### Env and Gag co-transfer to target cell through VS

**Fig. 7:**
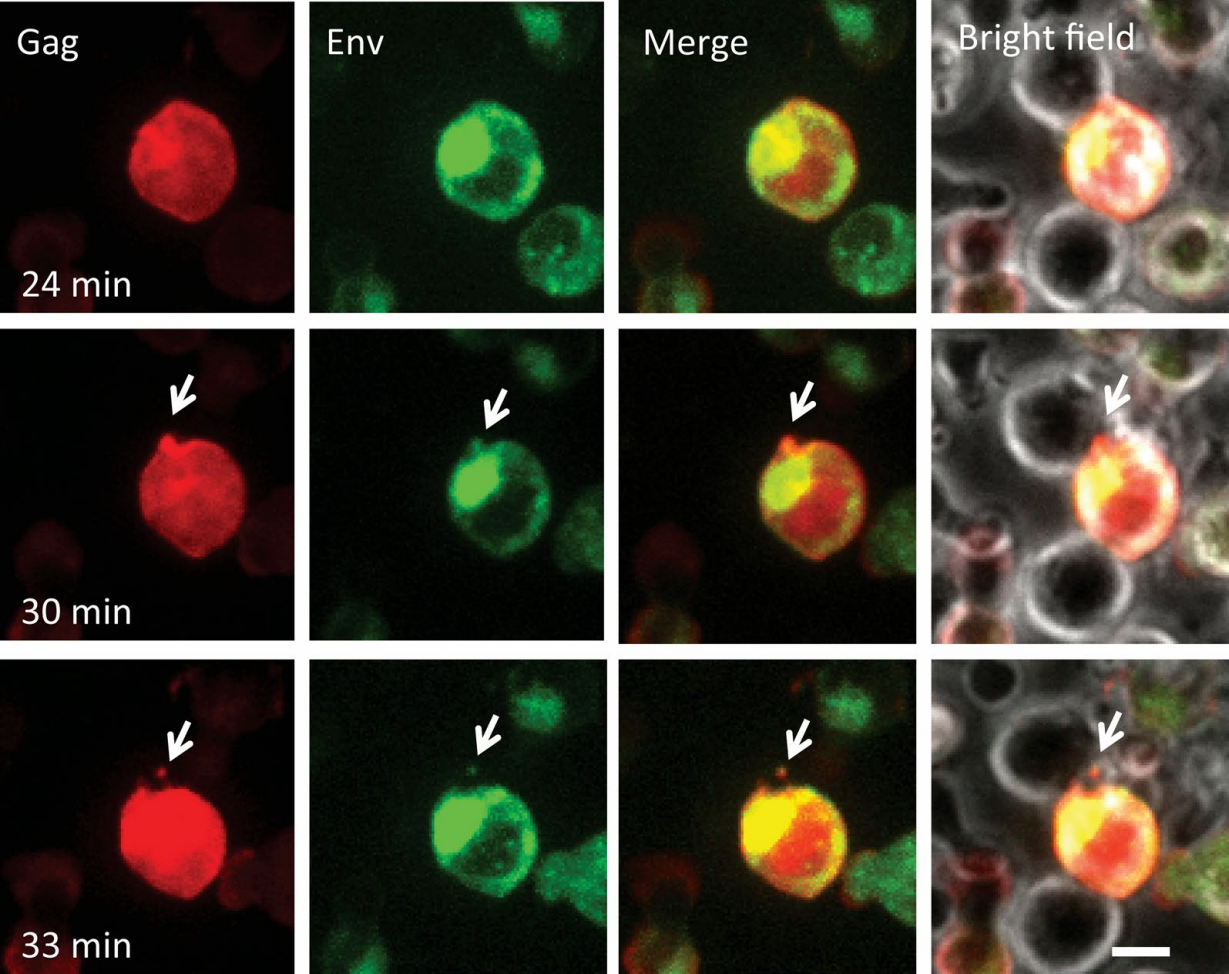
Live images of co-transfer of Gag and Env containing puncta from a donor cell to a target cell. Donor cells were Jurkat cells co-transfected with Gag-iCherry and Env-isfGFP-∆V1V2. Target cells, primary peripheral blood CD4 T cells, were mixed with donor cells at 24 h post transfection. Confocal z stacks were acquired at 3-min intervals from 0.5 h post co-culture for 3 h. Bar = 8 μm

Thus far live imaging studies have yet to capture the co-transfer of Gag and Env across VS. Co-expression of Gag-iCherry and Env-isfGFP-∆V1V2 in donor Jurkat T cells enabled simultaneous tracking of the recruitment of Gag and Env to the contact site between a donor and a target cell. At a frame rate of one per 5 min and imaging duration of 3.5 h, we followed the transfer of viral material across the VS with fluorescent Gag and Env (Fig. 7). The VS formation can be divided into the following three stages: (1) stable contact (top row), (2) formation of a synapse button with both Gag and Env (middle row) and (3) transfer of viral material (lower row) (Fig. 7). As compared to synapse formation, the transfer step occurred rapidly [6]. Using a faster frame rate of 1 frame per 1.2 s, an example of Gag and Env co-transfer is shown where a synaptic button between a donor and a target cell has already formed (Additional file 8: Movie S7). The co-transfer of Gag and Env to the target cell occurred within 2 min.

## Discussion

In this study, NL4-3 with a superfolder GFP fused Env (Env-isfGFP-∆V1V2) was constructed as a tool to observe Env during HIV-1 cell-to-cell spread. Transfection of Env-isfGFP-∆V1V2 failed to produce full-length Env protein, and displayed an unexpected defect in virus particle production. Co-transfection with a wild-type construct (or other HIV constructs with full length Gag DNA sequence, resulted in the rescue of the viral gene expression as well as the full-length Env-isfGFP-∆V1V2 protein. The underlying reason of this protein expression deficiency is unclear, co-expression of Gag-expressing proviral constructs appear to allow the production of cells and virus particles that display Env-isfGFP-∆V1V2 protein on the surface. Importantly, when Env-isfGFP-∆V1V2 was complemented, the entire viral protein profile was restored and wild-type amounts of viral particles were produced and enabled us to image the distribution of EnvisfGFP-∆V1V2, and its participation in cell–cell transfer events.

Using confocal imaging we found that Env-isfGFP-∆V1V2 protein was incorporated efficiently onto newly produced viral particles. These chimeric viruses were infectious but had lower infectivity. By co-transfecting Env-isfGFP-∆V1V2 with a CD4 binding site mutant NL4-3-D368R, we found that while the quantity of viral particles produced by D368R co-transfection was comparable to wild-type co-transfection (data not shown), the infectivity was lost in free viral particles produced by co-transfection with CD4-binding mutant, D368R. When viruses were produced by complementation with an NL4-3-L26R Env fusion peptide mutant and these also were not infectious (Fig. 2d). However, when the same Env-isfGFP-∆V1V2 and D368R co-transfectant was tested in cell-to-cell viral transfer, these were found to mediate greater viral antigen transfer compared to D368R mutant, indicating that some CD4 binding ability and synapse forming ability is retained in Env-isfGFP-∆V1V2 (Fig. 2e). The inability of the Env-isfGFP-∆V1V2 to rescue any infectivity shows that it likely to be incompatible with viral fusion.

When studied in cell-to-cell HIV transfer assays, Env-isfGFP-∆V1V2 protein was transferred across VSs in a CD4-dependent manner. Some transfer was observed even when the mutant Env was complemented with a CD4 binding site mutant. This indicated that the Env-isfGFP-∆V1V2 presents the requisite signals to participate in synapse-mediated viral transfer, despite its inferred lack of viral membrane fusion activity. The ability of Env-isfGFP-∆V1V2 to participate in the VS encouraged us to follow recruitment of Env-isfGFP-∆V1V2 to the VS using this system. Previous work has established that Env on the donor cell binding to CD4 on the target cell is required for the formation of VS [1, 5]. Furthermore, co-transfection of this construct with HIV Gag-iCherry, which has a wild type Env, allowed us to simultaneously track Gag and Env during synapse formation to interrogate the sequence of recruitment to the VS.

In our dual Gag and Env imaging studies, we found that as expected, the expression patterns of Env and Gag are very distinct. In previous studies, fluorescent Gag signal localizes prominently to the plasma membrane at the late stages of infection [2], while the majority of Env remains associated with internal trans Golgi network or recycling endosomes. Importantly, in Env-isfGFP-∆V1V2-expressing cells, we did not observe pre-accumulation of Env on cell surface prior to VS formation, but rather saw only low levels of accumulation to the contact area when a donor cell conjugated with a target cell (Fig. 5). This is consistent with descriptions of the low surface Env that is maintained by active endocytosis from the cell surface via AP-2 mediated clathrin-dependent pathways [22, 23]. Low Env exposure on the cell surface is likely to serve as an immune evasion strategy [24]. In live imaging studies we also found that when a Gag synaptic button was already formed, fluorescent Env was not uniformly or persistently observed at the synapse. This could be a limitation of the fluorescent Env which gets displaced over time by wild type Env at the synapse, or it could be reflective of the true physiology. Other labeling approaches will need to be developed to distinguish between these alternatives. The recruitment of Env to the synapse in the formation stage appears to be a highly dynamic process and may be influenced by recycling of Env from the surface [25, 26]. Env-isfGFP-∆V1V2 gives us a picture of the overall distribution of Env in transfected cells, and indicates that levels of Env at the cell surface are low. Given the low levels of Env we observe at the VS, it is possible that the Env molecules only accumulate at the contact area transiently and that only a small number of molecules are required to initiate the VS. Adhesion molecules that can participate in the VS may help stabilize cell–cell interactions so that high levels of Env are not required [27–29]. Env that is not involved in virus particle assembly may continue to be internalized from the surface [24, 30], leading to low levels of Env at the VS that are not easily detected. The recycling pools of Env at the VS may be important for efficient incorporation of Env onto the newly formed virus particles [25].

**Fig. 8:**
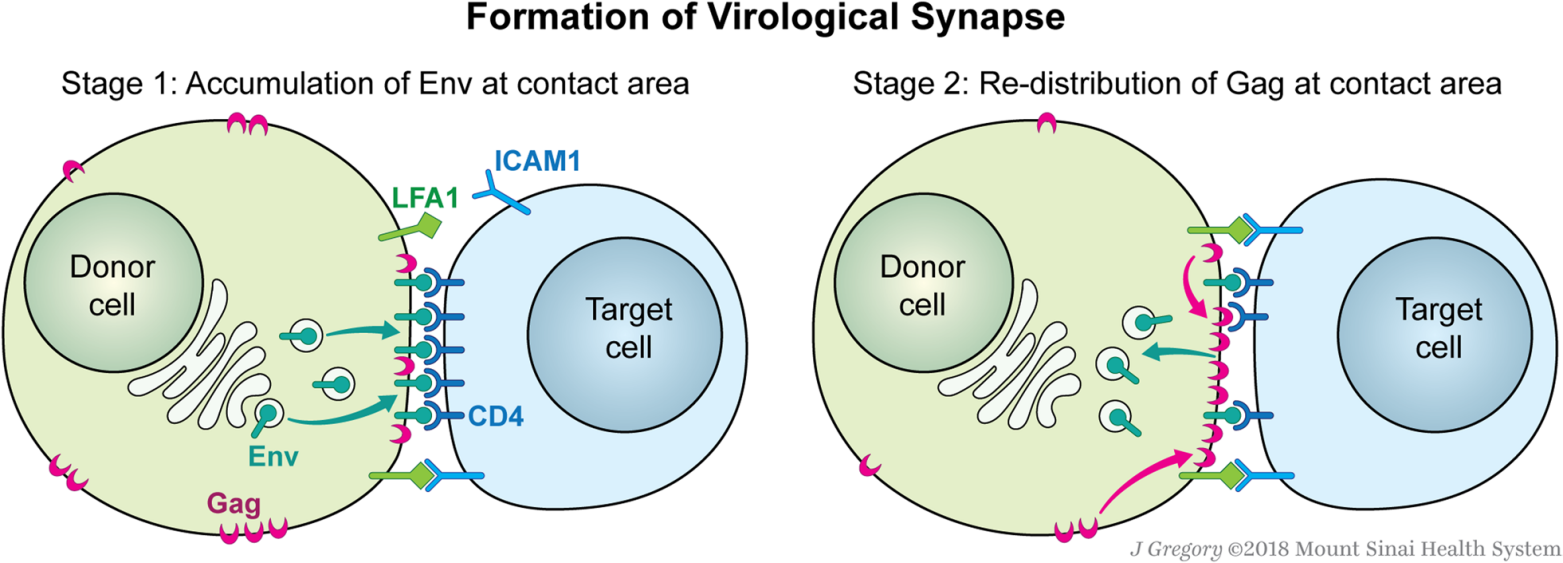
A two-step model of HIV-1 VS formation. Based on time-lapse fluorescence microscopy, formation of HIV-1 VS following a two-step process. At stage 1, Env accumulation is induced at the site of cell contact between an HIV infected donor cell and an uninfected target CD4 cell. Env on the donor cell interact with CD4 on target cell to stabilize the contact. Other adhesive molecules, e.g. ICAM-1, may also play a role in the formation of stable contact. At stage 2, after a stable contact has been established, Gag redistributes at the contact area to form a “button” like structure

The observations presented here support a sequential recruitment model where an Env dependent adhesion process and low levels of Env accumulation precede Gag recruitment (Fig. 8). In this model, there are two stages of VS formation. The first step is the attachment of donor and target cells when Env serves an important role as an inducer of cell–cell adhesion, prior to its incorporation into the virus particle. Donor and target cells may contact each other spontaneously or may be attracted by chemokines or stabilized by adhesion molecules to facilitate attachment. Attachment is accomplished without a requirement for a large pre-accumulated source of Env at the contact site and the Env trafficking is subsequently directed to the attachment site. Live imaging studies indicated that Env accumulation occurred rapidly: often within 10 min of the initial adhesion (Fig. 5 and Additional file 1: Table S1). Env can interact with CD4 on the target cell and stabilize the contact or alternatively the interaction can be short-lived where the donor and target cells detach. In Fig. 5d, events associated with Env accumulation duration ≤ 10 min were the result of detachment of donor and target cells. It will be interesting to study the role of other cellular adhesion molecules implicated in regulating the HIV-1 VS in promoting or diminishing these initial interactions [1, 27, 29, 31]. The second stage of VS formation is the re-distribution of Gag. Prior studies have shown that Gag can accumulate in a polarized manner even without Env [32, 33]. However, during synapse formation, we observe that Gag redistribution to cell contact area occurs after Env recruitment (Fig. 6). Transfer of particles containing Gag and Env at VS is a very late event, which is likely to be the result of coordinated assembly and budding of newly formed virus particles.

## Conclusions

Live imaging of Env-isfGFP-∆V1V2 supports a sequential model of Env and Gag recruitment to the VS. In this model, Env and Gag follow independent sequential pathways to form a VS. Low levels of Env can accumulate at the cell contact site within minutes of cell–cell contact, while higher levels of Gag redistribute to the same established site of cell–cell contact afterwards to form a button-like structure. Both Env and Gag can be observed to co-transfer to target cell simultaneously. These studies reinforce the notion that Env is acting initially as a cell adhesion molecule prior to performing its essential functions in mediating viral membrane fusion.

## Methods

### Viral constructs

HIV-1 Gag-iGFP is a molecular clone based on pNL4-3 [34] with green fluorescent protein (GFP) inserted between the Gag MA and CA domains [2]. Gag-iCherry is the same as Gag-iGFP but with mCherry in place of GFP. HIV Env-∆V1V2-isfGFP is an NL4-3-based HIV-1 molecular clone that carries superfolder green fluorescent protein (sfGFP) inserted internally into Env to substitute V1V2 domain. A CD4 binding site mutation D368R was introduced into NL4-3 to form NL4-3-D368R [18]. Gag-iCherry-D368R is also CD4 binding site mutant but the backbone is Gag-iCherry. A fusion peptide point mutation L26R was introduced into NL4-3 to form NL4-3-L26R [17].

### Cell culture and production of viruses

The CD4 T cell line Jurkat CE6.1 (ATCC) was maintained in RPMI 1640 with 100 U/ml penicillin and 100 µg/ml streptomycin plus 10% fetal bovine serum (FBS). Cells were passaged regularly and were maintained at concentrations of less than 10^6^/ml. Primary CD4^+^ T cells were obtained from human peripheral blood through the New York Blood Center and isolated by negative selection with a Miltenyi CD4 T cell isolation kit II (Miltenyi Biotec). Unactivated CD4 T cells were maintained in complete RPMI medium containing 50 U/ml interleukin 2 (IL-2; ARP). Cell-free virus was produced by transfection of 293T cells using calcium phosphate method with 20 μg of viral plasmid per 10 cm plate. Media was exchanged 16 h after transfection and virus supernatants were harvested 48 h after transfection.

### p24 ELISA

The p24 ELISA was performed by a modified version of a previously published protocol. Costar 3922 flat-bottomed, high binding plates were incubated at room temperature with anti-p24 antibody overnight (Aalto D7320; 1:200 in 0.1 M NaHCO_3_). The plate was washed twice with 1× TBST and blocked with 2% nonfat dry milk (Lab Scientific) for 1 h then washed in TBST. HIV supernatants treated with 1% Empigen (1:100 and 1:1000 in DMEM) along with titration of p24 standard are added to wells and incubated at room temperature for 2 h, then washed 4× with TBST. Alkaline phosphatase conjugated mouse anti-HIV p24 (CLINIQA) was added (1:8000 in TBST 20% sheep serum) and incubated for 1 h followed by 6 TBST washes. 50μl of Sapphire Substrate (Tropix) was added to each well and incubated for 20 min. Luminescence was quantitated on Fluo Star Optima plate reader and sample values calculated based on nonlinear regression of standard curve using Prism software (Graphpad software inc.).

*Western blot analysis* 293T cells were transfected using homemade PEI Transfection reagent (Sigma) with 3 μg of plasmid DNA per well of 6 well plate. Cells were lysed with RAPA and protease inhibitor cocktail (Sigma). Lysate equivalent of approximately 2 × 105 cells per well were run on NuPage 4–12% Bis–Tris Gel (Novex) and transferred to Amersham Hybond-P PVDF membranes (GE Healthcare). Membranes were blocked with 2% nonfat dry milk (Lab Scientific), then probed with rabbit anti-GFP (1:5000), human anti-HIV serum (1:10,000) primary antibodies then anti-rabbit (Jackson Immunoresearch) or anti-human horseradish peroxidase (Jackson Immunoresearch) conjugated secondary antibody.

### TZM-bl assay

Cell-free viruses were produced in 293T cells. TZM-bl cells were plated at 2 × 104 cells/well in 96 well plates and incubated at 37 °C with indicated viruses. Media was replaced after 24 h of infection and incubated for another 24 h. At 48 h postinfection, Media was aspirated followed by lysis in Luciferase Cell Culture Lysis Reagent (Promega). 20 μl of each sample was read on Fluo Star Optima plate reader with an injection of 50 μl of Luciferase Assay Reagent (Promega).

### Cell-to-cell transfer assay

HIV-1 proviral constructs were transduced into Jurkat cells (donor cells) using Amaxa nucleofection as previously described (Amaxa Biosystems). In brief, 5 μg of endotoxin-free HIV-1 proviral plasmids was nucleofected into 6 × 10^6^ Jurkat cells using Cell Line Nucleofector kit V, program S-18. Twenty hours after nucleofection, viable Jurkat cells were purified by centrifugation on a Ficoll-Hypaque density gradient. Twenty-four hours after nucleofection, cells were washed with complete buffer and recovered at 37 °C for co-culture. Unactivated primary CD4^+^ T cells (target cells) were resuspended in serum-free RPMI 1640 and stained with 1 μM Cell-Tracker Orange CMTMR (5-and-6-(((4chloromethyl)benzoyl)amino)tetramethylrhodamine); Molecular Probes) for 45 min at 37 °C, washed, and cultured overnight in complete RPMI medium containing 50 U/ml IL-2. Donor and target cells were mixed at a ratio of approximately 1:1 and cocultured at 37 °C for 3 h before they were treated with trypsin and fixed. Where inhibitor Leu3a, an HIV-blocking anti-CD4 antibody (BD Biosciences) was used, donor and target cells were preincubated separately with equal volumes of inhibitor for 30 min at 37 °C before mixing.

### Fluorescence microscopy sample preparation

Transfected Jurkat cells (donor cells) were mixed with primary CD4 cells (target cells) in round bottom 96-well-plates for 3–4 h as previously described [5]. Pipette tips were trimmed to reduce the shearing to cells. Co-cultured donor and target cells were carefully transferred without disturbance onto poly-lysine treated coverslips. The cells were kept on the coverslip for 30 min in 37 °C incubator to attach to the poly-l-lysine treated surface. Then media was carefully aspirated and cells were fixed with 4% PFA for at least 10 min at room temperature, washed twice with PBS and stained with anti-fade mounting medium with DAPI (Vectashield, Co#: H-1200, Vector Laboratories). Coverslides were sealed with nail polish and the samples were observed under fluorescent microscope.

### Confocal and live imaging

Confocal imaging was carried out on an inverted Leica SP5 DMI laser scanning confocal microscope, using a 63× objective and analyzed using Volocity (PerkinElmer) or ImageJ (NIH) software. Live imaging was carried out in a sealed but gas permeable microchamber slides (µ-Slide VI ^0.4^, ibidi Biosciences). Nucleofected Jurkat cells were used for overnight expression of Env. For imaging of HIV cell-to-cell transfer, donor cells are nucleofected Jurkat cells and target cells are primary CD4 T cells or MT4 cells. Donor cells were mixed with target cells at a ratio of 1:2 and were loaded onto the micro-chamber pre-coated with 150 g/ml fibronectin to provide the cells with a two-dimensional substrate for attachment and migration. The chamber was placed on a Zeiss AxioObserver Z1 inverted microscope mounted with Yokogawa CSU-X1 spinning disk scan head. Dual Hamamatsu EM-CCD C9100 digital cameras enable simultaneous imaging of up to two fluorescent channels. Phase contrast imaging and confocal green (for sfGFP) and red (for mCherry) fluorescence were acquired in a multitrack configuration to avoid cross-talk between fluorescence channels. Images were recorded at different time intervals continuously as indicated in results. Confocal images and Quicktime movies were generated from laser-scanning confocal microscope file data using Metamorph software (Molecular Devices) and Imaris (bitmap) software.

### Env-isfGFP-∆V1V2 protein structure modeling

A green fluorescence protein (GFP) molecule with a terminal disulfide bond was generated using ROSETTA software [35]. Briefly, full-length GFP sequence was first threaded on a GFP crystal structure (PDB ID 4EUL) [36], and its N- and C-terminal residues that were not resolved in the crystal structure modeled and protein backbone fragments from gp120 residues 123–127 and 196–199 (obtained from the SOSIP structure; PDB ID 4TVP [10]) were then inserted onto the N- and C-termini, respectively, of the threaded GFP structure using the minirosetta comparative modeling protocol. During the modeling protocol distance constraints were imposed between Cβ atoms and between SG atoms of the cysteine residues on which the disulfide bond was to be built. The final SOSIP-GFP models were generated in Pymol [37] by superimposing the disulfide bond in the lowest energy homology GFP model onto the corresponding V1V2 disulfide bond of the 3.5 Å resolution BG505 SOSIP.664 structure (PDB ID 4TVP) [10].

## Additional files

**Additional file 1: Fig. S1.** Env-isfGFP-ΔV1V2 expression in 293T cells. **Fig. S2.** Env-ΔV1V2-isfGFP complemented with wild type Env HIV-1 constructs: surface stain of Env with CD4 binding site antibody b12 (A) or anti-GFP (B). **Fig. S3.** Examples of sequential Env and Gag accumulation during VS formation. **Table S1.** Summary of contact-induced accumulation of Env at sites of cell-cell contact. Cell counts and interactions enumerated in five fields of view during Env-isfGFP-ΔV1V2 overnight expression in Jurkat cells. Continuous imaging performed over 32 h was acquired at 10-min intervals. **Table S2.** Cell counts and interactions enumerated in four fields of view during cell-to-cell HIV infection. Gag-iCherry and Env-ΔV1V2-isfGFP co-transfected Jurkat cells were mixed with primary CD4 target cells 24 h post nucleofection. Continuous imaging over 3 h acquired at 3-min intervals.

**Additional file 2: Movie S1.** De-novo expression of sfGFP Env in Jurkat cell. Live time-lapse confocal fluorescence imaging of an Env-isfGFP-ΔV1V2-expressing Jurkat lymphoblastoid T cell. Confocal z stacks were acquired at 10-min intervals starting at 5 h post transfection. A representative cell is selected here and the sharpest layer of the image stack is displayed. The cell migrated out of the field of view at 26 h post transfection.

**Additional file 3: Movie S2.** Env accumulation at sites of cell-cell contact. In this example, Env accumulates at the site of cell-cell contact, beginning within 10 min after contact. Env accumulation increases at 20 min after contact. The white arrow indicates the position where Env accumulates. Images were recorded every 10 min using Dual Hamamatsu EM-CCD C9100 digital cameras with Yokogawa CSU-X1 spinning disk scan head. Z dimension is acquired continuously with 17 steps covering 25 μm and the sharpest layers are shown here. Duration of this movie is 1 h.

**Additional file 4: Movie S3.** Gag is active and abundant at the leading edge of Gag-iCherry and Env-ΔV1V2-isfGFP co-transfected Jurkat cells. A paused frame shows abundant Gag at the leading edge, where no Env accumulation was detected. Images were recorded every 8 s using Dual Hamamatsu EM-CCD C9100 digital cameras with Yokogawa CSU-X1 spinning disk scan head. Only the sharpest single focal planes are shown in the movie.

**Additional file 5: Movie S4.** Live imaging shows a synapse where several Env puncta are localized to the cell-cell contact site before Gag redistribution to the VS. Jurkat cells were co-transfected with Gag-iGFP and Env-isfGFP-ΔV1V2 as donor cells. A paused frame shows the Env localized at cell contact area before a Gag button formed. A false color lookup table view of Env reveals the Env puncta. Target cells were primary human CD4 T cells. Images were recorded every 10 s using Dual Hamamatsu EM-CCD C9100 digital cameras with Yokogawa CSU-X1 spinning disk scan head. Z dimension was acquired continuously with 18 steps and the sharpest focal planes are displayed here.

**Additional file 6: Movie S5.** A transient Env accumulation is observed before Gag “button” is formed during a forming VS. Images were recorded every 3 min using a widefield microscope. The white arrowhead shown in each channel highlights a putative forming synapse. The paused frame shows accumulated Env at t = 6 min when Gag also became obvious at cell-cell contact. Z dimension was acquired continuously with 10 steps covering 15 μm and the sharpest focal planes are shown in the movie. RLT: reference lookup table; bar: 5 μm.

**Additional file 7: Movie S6.** Live imaging of formed polysynapses on a donor cell. The paused frame shows minimal Env accumulated at the contact sites where five Gag buttons are already observed. Jurkat cells were co-transfected with Gag-iGFP and Env-isfGFP-ΔV1V2 as donor cells. Target cells were primary human CD4 T cells. Images were recorded every 1.6 s using a Dual Hamamatsu EM-CCD C9100 digital cameras with Yokogawa CSU-X1 spinning disk scan head. Z dimension was acquired continuously with 10 steps. Duration of this movie is 5 min and 48 s.

**Additional file 8: Movie S7.** Live cell imaging showing transfer of both Gag and Env across a virological synapse. Jurkat cells were co-transfected with Gag-iGFP and Env-isfGFP-ΔV1V2 as donor cells. Target cells were primary human CD4 T cells. A paused frame highlights Env with a white arrowhead at the site where Gag transfer is also apparent. Images were recorded every 1.2 s using a Dual Hamamatsu EM-CCD C9100 digital cameras with Yokogawa CSU-X1 spinning disk scan head. Z dimension was acquired continuously with 7 steps and the sharpest focal planes are shown. The movie duration is 1 min and 56 s.

## Abbreviations

VS: virological synapse
sfGFP: superfolder GFP
RLT: reference lookup table
