# Supplementary material for: Sequential trafficking of Env and Gag to HIV-1 T cell virological synapses revealed by live imaging
Source: Retrovirology. 2019 Jan 15;16:2. doi: 10.1186/s12977-019-0464-3 (PMC6334456; doi:10.1186/s12977-019-0464-3)
Supplement: Supplementary file 1 — Additional file 1: Fig. S1. Env-isfGFP-ΔV1V2 expression in 293T cells. Fig. S2. Env-ΔV1V2-isfGFP complemented with wild type Env HIV-1 constructs: surface stain of Env with CD4 binding site antibody b12 (A) or anti-GFP (B). Fig. S3. Examples of sequential Env and Gag accumulation during VS formation. Table S1. Summary of contact-induced accumulation of Env at sites of cell-cell contact. Cell counts and interactions enumerated in five fields of view during Env-isfGFP-ΔV1V2 overnight expression in Jurkat cells. Continuous imaging performed over 32 h was acquired at 10-min intervals. Table S2. Cell counts and interactions enumerated in four fields of view during cell-to-cell HIV infection. Gag-iCherry and Env-ΔV1V2-isfGFP co-transfected Jurkat cells were mixed with primary CD4 target cells 24 h post nucleofection. Continuous imaging over 3 h acquired at 3-min intervals. [file 12977_2019_464_MOESM1_ESM.docx]

**Supplementary Data**

Figure. S1: Env-isfGFP-∆V1V2 expression in 293T cells.


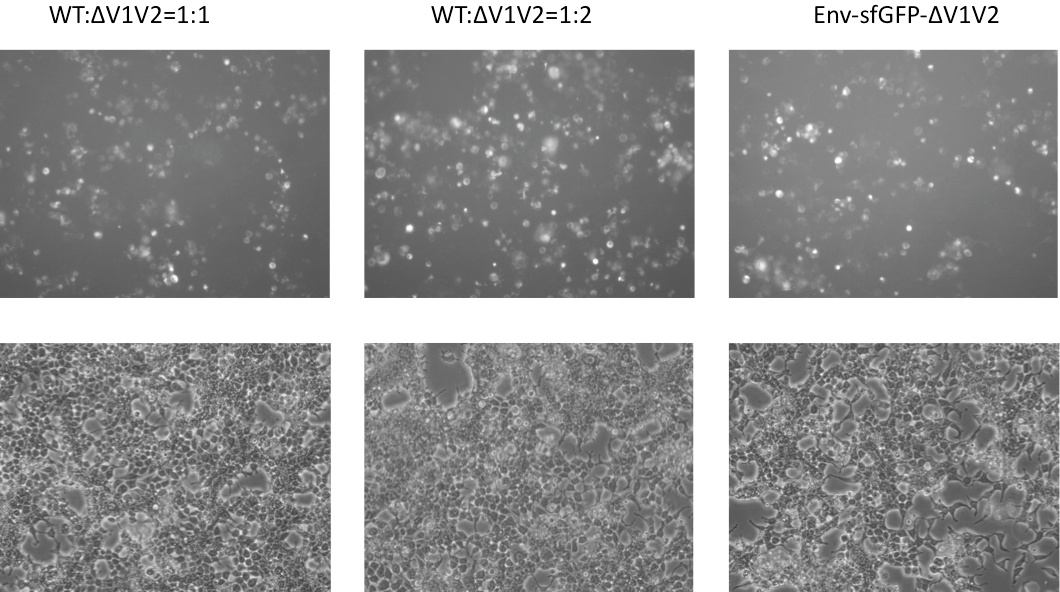


Fig. S2: Env-∆V1V2-isfGFP complemented with wild type Env HIV-1 constructs: Surface stain of Env with CD4 binding site antibody b12 (A) or anti-GFP (B).


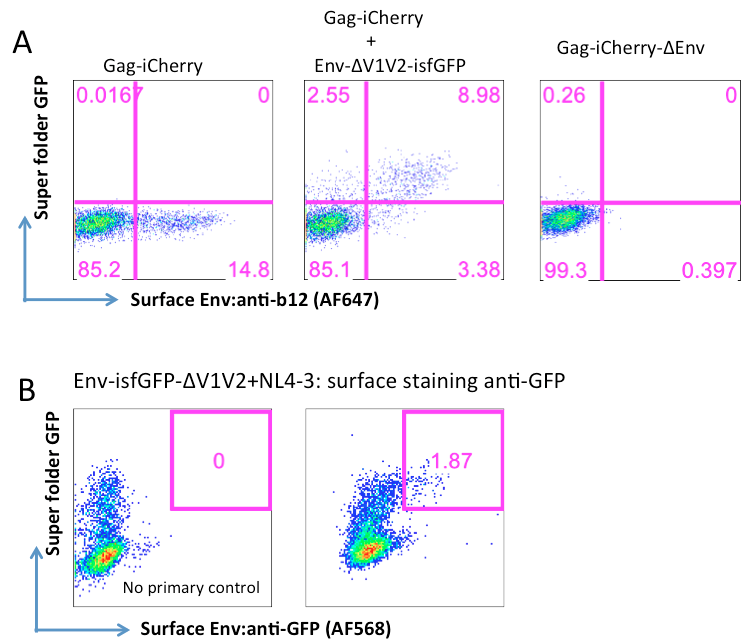


Fig. S3: Examples of sequential Env and Gag accumulation during VS formation.


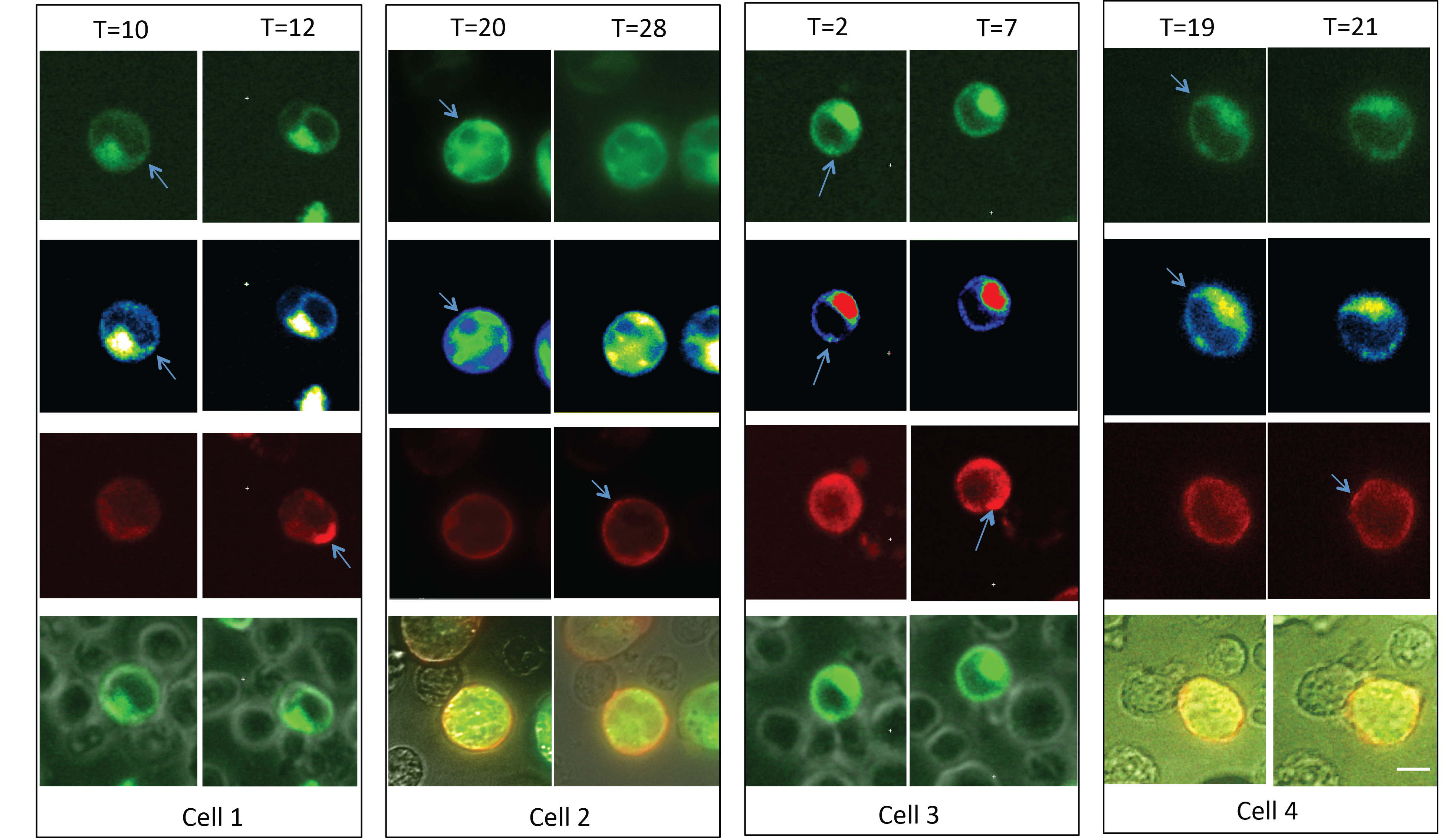


Table S1: Summary of contact-induced accumulation of Env at sites of cell-cell contact. Cell counts and interactions enumerated in 5 fields of view during Env-isfGFP-∆V1V2 overnight expression in Jurkat cells. Continuous imaging performed over 32 hours was acquired at 10-minute intervals.

| Tracked HIV+ donor cells | Donor cells have contact with target cells | Donor cells have prolonged contact with putative target cells  Start time Duration  (minute) (minutes) | | Donor cells with surface Env accumulation | Long term contact with Env accumulation |
| --- | --- | --- | --- | --- | --- |
| \| 1-01 \| \| --- \| \| 1-02 \| \| 1-03 \| \| 1-04 \| \| 1-05 \| \| 1-06 \| \| 1-07 \| \| 1-08 \| \| 1-09 \| \| 1-10 \| \| 1-11 \| \| 1-12 \| \| 1-13 \| \| 2-01 \| \| 2-02 \| \| 2-03 \| \| 2-04 \| \| 2-05 \| \| 2-06 \| \| 2-07 \| \| 2-08 \| \| 3-01 \| \| 3-02 \| \| 3-03 \| \| 3-04 \| \| 3-05 \| \| 3-06 \| \| 3-07 \| \| 4-01 \| \| 4-02 \| \| 4-03 \| \| 4-04 \| \| 4-05 \| \| 4-06 \| \| 4-07 \| \| 4-08 \| \| 4-09 \| \| 4-10 \| \| 5-01 \| \| 5-02 \| \| 5-03 \| \| 5-04 \| \| 5-05 \| \| 5-06 \| \| 5-07 \| | \| × \| \| --- \| \| × \| \| × \| \| × \| \| × \| \| × \| \| × \| \| × \| \| × \| \| × \| \| × \| \| × \| \| × \| \| × \| \| × \| \| × \| \| × \| \| × \| \| × \| \| × \| \| × \| \| × \| \| × \| \| × \| \| × \| \| × \| \| × \| \| × \| \| × \| \| × \| \| × \| \| × \| \| × \| \| × \| \| × \| \| × \| \| × \| \| × \| \| × \| \| × \| \| × \| \| × \| \| × \| \| × \| \| × \| | \|  \| \| --- \| \|  \| \| 10 \| \|  \| \|  \| \|  \| \|  \| \| 900 \| \| 450 \| \|  \| \|  \| \| 930 \| \| 640 \| \| 540 \| \|  \| \|  \| \|  \| \|  \| \|  \| \|  \| \|  \| \|  \| \|  \| \|  \| \|  \| \|  \| \| 1210 \| \|  \| \|  \| \|  \| \|  \| \| 760 \| \|  \| \|  \| \|  \| \| 1200 \| \|  \| \|  \| \|  \| \| 1540 \| \| 1100 \| \|  \| \| 0 \| \|  \| \| 910 \| | \|  \| \| --- \| \|  \| \| 1730 \| \|  \| \|  \| \|  \| \|  \| \| 160 \| \| 1300 \| \|  \| \|  \| \| 820 \| \| 1110 \| \| 490 \| \|  \| \|  \| \|  \| \|  \| \|  \| \|  \| \|  \| \|  \| \|  \| \|  \| \|  \| \|  \| \| 410 \| \|  \| \|  \| \|  \| \|  \| \| 990 \| \|  \| \|  \| \|  \| \| 550 \| \|  \| \|  \| \|  \| \| 210 \| \| 650 \| \|  \| \| 1750 \| \|  \| \| 840 \| | \|  \| \| --- \| \|  \| \| × \| \| × \| \|  \| \| × \| \|  \| \| × \| \| × \| \| × \| \|  \| \| × \| \| × \| \| × \| \| × \| \|  \| \| × \| \|  \| \| × \| \| × \| \| × \| \| × \| \| × \| \| × \| \| × \| \|  \| \| × \| \|  \| \| × \| \| × \| \| × \| \| × \| \| × \| \| × \| \|  \| \| × \| \| × \| \|  \| \|  \| \| × \| \| × \| \| × \| \| × \| \| × \| \| × \| | \|  \| \| --- \| \|  \| \| × \| \|  \| \|  \| \|  \| \|  \| \| × \| \|  \| \|  \| \|  \| \| × \| \| × \| \| × \| \|  \| \|  \| \|  \| \|  \| \|  \| \|  \| \|  \| \|  \| \|  \| \|  \| \|  \| \|  \| \| × \| \|  \| \|  \| \|  \| \|  \| \|  \| \|  \| \|  \| \|  \| \| × \| \|  \| \|  \| \|  \| \| × \| \| × \| \|  \| \| × \| \|  \| \| × \| |
| total | 45 |  |  | 33 | 11 |

Table S2: Cell counts and interactions enumerated in four fields of view during cell-to-cell HIV infection. Gag-iCherry and Env-∆V1V2-isfGFP co-transfected Jurkat cells were mixed with primary CD4 target cells 24 hours post nucleofection. Continuous imaging over 3 hours acquired at 3-minute intervals.

| **Tracked HIV+ donor cells** | **Prolonged donor cell contact with target cells (≥12 min)** | **Donor: Target contact start time and duration** | | **Donor cells with surface Env accumulation** | **Donor cells with both Env and Gag at cell contact** |
| --- | --- | --- | --- | --- | --- |
|  |  | **Start time Duration**  **(minute) (minutes)** | |  |  |
| 1-01 | x | 0 | 174 |  |  |
| 1-02 | x | 102 | 65 | x |  |
| 1-03 | x | 0 | 174 | x | x |
| 1-04 |  |  |  |  |  |
| 1-05 |  |  |  |  |  |
| 1-06 |  |  |  |  |  |
| 1-07 | x | 12 | 162 | x | x |
| 1-08 | x | 0 | 174 | x |  |
| 1-09 | x | 63 | 110 | x | x |
| 1-10 | x | 15 | 159 | x | x |
| 1-11 |  |  |  | x |  |
| 1-12 | x | 0 | 174 |  |  |
| 1-13 |  |  |  | x |  |
| 2-01 |  |  |  |  |  |
| 2-02 | x | 42 | 122 | x | x |
| 2-03 | x | 0 | 174 |  | x |
| 2-04 | x | 0 | 174 |  | x |
| 2-05 |  |  |  |  |  |
| 2-06 |  |  |  |  |  |
| 2-07 |  |  |  |  |  |
| 2-08 |  |  |  |  |  |
| 2-09 | x | 0 | 174 | x |  |
| 2-10 | x | 120 | 54 | x | x |
| 3-2-01 |  |  |  |  |  |
| 3-2-02 | x | 0 | 129 | x |  |
| 3-2-03 | x | 0 | 129 | x | x |
| 3-2-04 | x | 0 | 129 | x | x |
| 3-2-05 |  | 117 | 12 | x |  |
| 3-2-06 |  | 84 | 30 | x |  |
| 3-2-07 | x | 0 | 129 | x | x |
| 3-2-08 | x | 0 | 129 | x | x |
| 3-2-09 |  | 18 | 12 | x |  |
| 3-2-10 |  |  |  |  |  |
| 5-01 | x | 0 | 183 | x | x |
| 5-02 | x | 33 | 150 | x | x |
| 5-03 | x | 66 | 115 | x | x |
| 5-04 |  |  |  |  |  |
| 5-05 |  |  |  | x |  |
| 5-06 |  |  |  |  |  |
| 5-07 | x | 54 | 129 | x | x |
| 5-08 | x | 0 | 183 |  |  |
| 5-09 |  |  |  |  |  |
| 5-10 | x | 60 | 123 | x |  |
| 5-11 |  |  |  |  |  |
| 5-12 |  |  |  |  |  |

Supplementary Movie S1: *de-novo* expression of sfGFP Env in Jurkat cell. Live time-lapse confocal fluorescence imaging of an Env-isfGFP-∆V1V2-expressing Jurkat lymphoblastoid T cell. Confocal z stacks were acquired at 10-min intervals starting at 5 hours post transfection. A representative cell is selected here and the sharpest layer of the image stack is displayed. The cell migrated out of the field of view at 26 hours post transfection.

Supplementary Movie S2: Env accumulation at sites of cell-cell contact. In this example, Env accumulates at the site of cell-cell contact, beginning within 10 minutes after contact. Env accumulation increases at 20 minutes after contact. The white arrow indicates the position where Env accumulates. Images were recorded every 10 minutes using Dual Hamamatsu EM-CCD C9100 digital cameras with Yokogawa CSU-X1 spinning disk scan head. Z dimension is acquired continuously with 17 steps covering 25 µm and the sharpest layers are shown here. Duration of this movie is 1 hour.

Supplementary Movie S3: Gag is active and abundant at the leading edge of Gag-iCherry and Env-∆V1V2-isfGFP co-transfected Jurkat cells. A paused frame shows abundant Gag at the leading edge, where no Env accumulation was detected. Images were recorded every 8 seconds using Dual Hamamatsu EM-CCD C9100 digital cameras with Yokogawa CSU-X1 spinning disk scan head. Only the sharpest single focal planes are shown in the movie.

Supplementary Movie S4. Live imaging shows a synapse where several Env puncta are localized to the cell-cell contact site before Gag redistribution to the VS. Jurkat cells were co-transfected with Gag-iGFP and Env-isfGFP-∆V1V2 as donor cells. A paused frame shows the Env localized at cell contact area before a Gag button formed. A false color lookup table view of Env reveals the Env puncta. Target cells were primary human CD4 T cells. Images were recorded every 10 seconds using Dual Hamamatsu EM-CCD C9100 digital cameras with Yokogawa CSU-X1 spinning disk scan head. Z dimension was acquired continuously with 18 steps and the sharpest focal planes are displayed here.

Supplementary Movie S5: A transient Env accumulation is observed before Gag “button” is formed during a forming VS. Images were recorded every 3 minutes using a widefield microscope. The white arrowhead shown in each channel highlights a putative forming synapse. The paused frame shows accumulated Env at t=6 min when Gag also became obvious at cell-cell contact. Z dimension was acquired continuously with 10 steps covering 15 µm and the sharpest focal planes are shown in the movie. RLT: reference lookup table; bar: 5 µm.

Supplementary Movie S6. Live imaging of formed polysynapses on a donor cell. The paused frame shows minimal Env accumulated at the contact sites where five Gag buttons are already observed. Jurkat cells were co-transfected with Gag-iGFP and Env-isfGFP-∆V1V2 as donor cells. Target cells were primary human CD4 T cells. Images were recorded every 1.6 second using a Dual Hamamatsu EM-CCD C9100 digital cameras with Yokogawa CSU-X1 spinning disk scan head. Z dimension was acquired continuously with 10 steps. Duration of this movie is 5 minutes and 48 seconds.

Supplementary Movie S7. Live cell imaging showing transfer of both Gag and Env across a virological synapse. Jurkat cells were co-transfected with Gag-iGFP and Env-isfGFP-∆V1V2 as donor cells. Target cells were primary human CD4 T cells. A paused frame highlights Env with a white arrowhead at the site where Gag transfer is also apparent. Images were recorded every 1.2 second using a Dual Hamamatsu EM-CCD C9100 digital cameras with Yokogawa CSU-X1 spinning disk scan head. Z dimension was acquired continuously with 7 steps and the sharpest focal planes are shown. The movie duration is 1 minute and 56 seconds.
